# Supplementary figures and images for: HPV-driven heterogeneity in cervical cancer: study on the role of epithelial cells and myofibroblasts in the tumor progression based on single-cell RNA sequencing analysis
Source: PeerJ. 2024 Sep 25;12:e18158. doi: 10.7717/peerj.18158 (PMC11438433; doi:10.7717/peerj.18158)

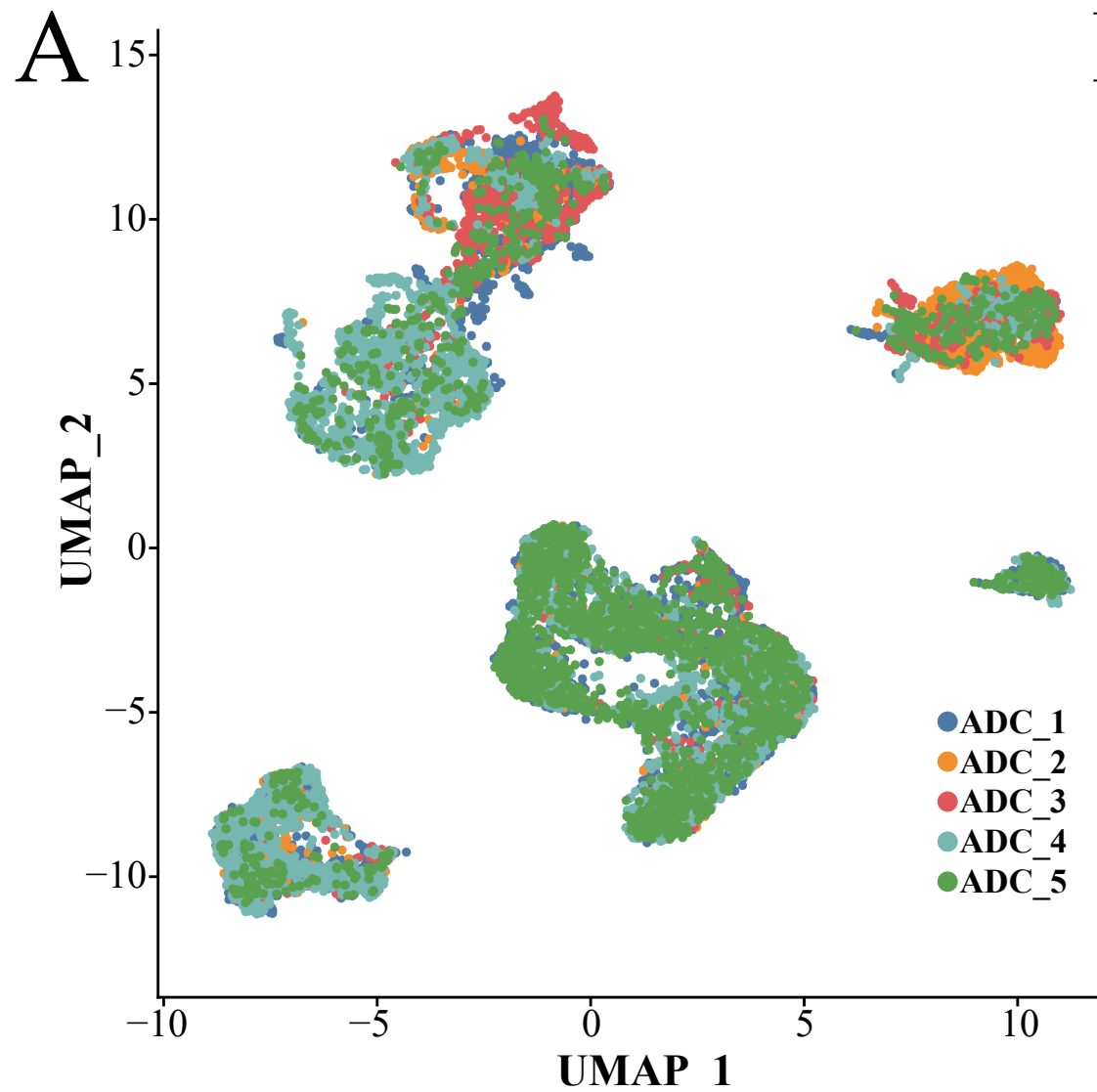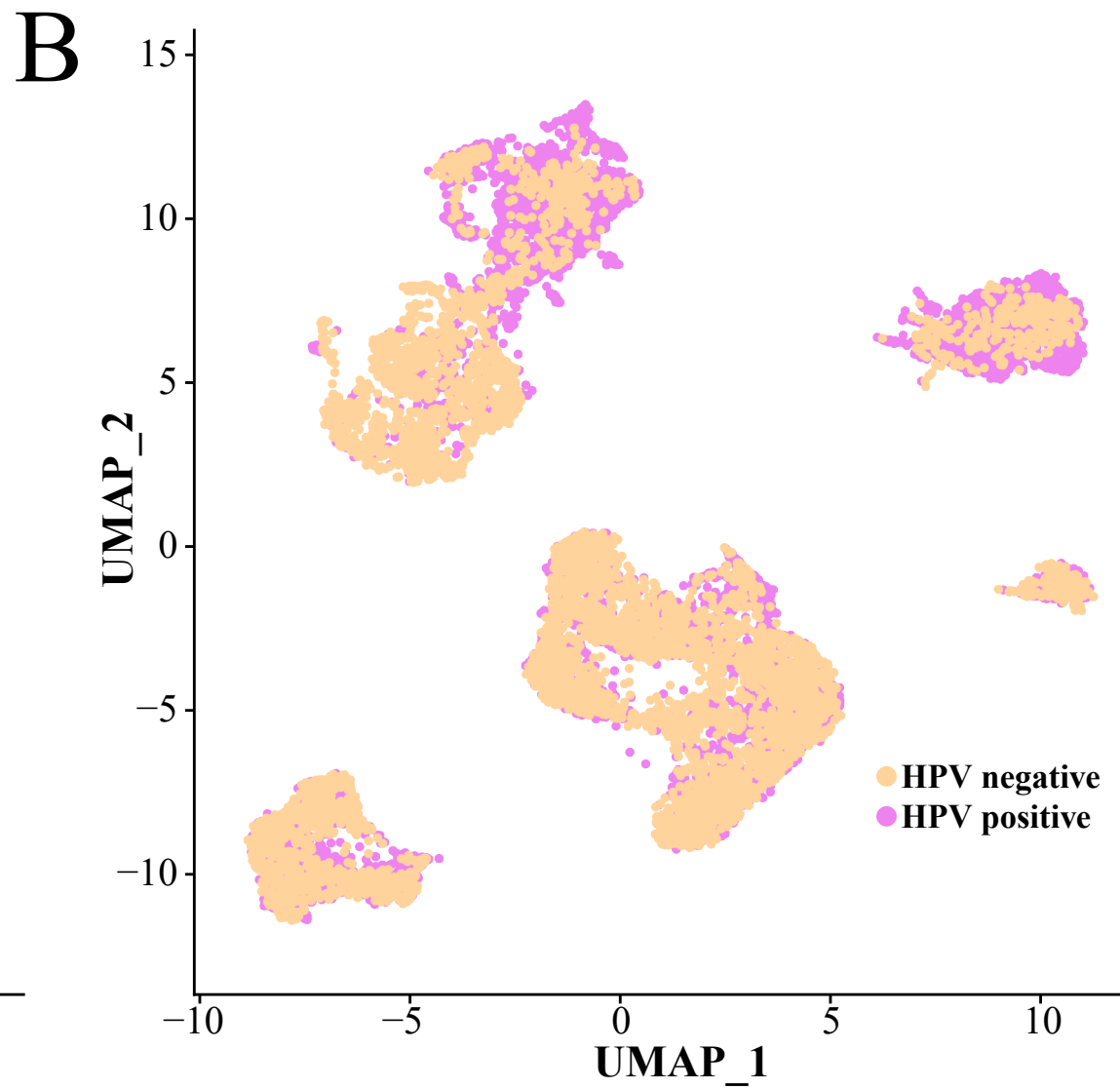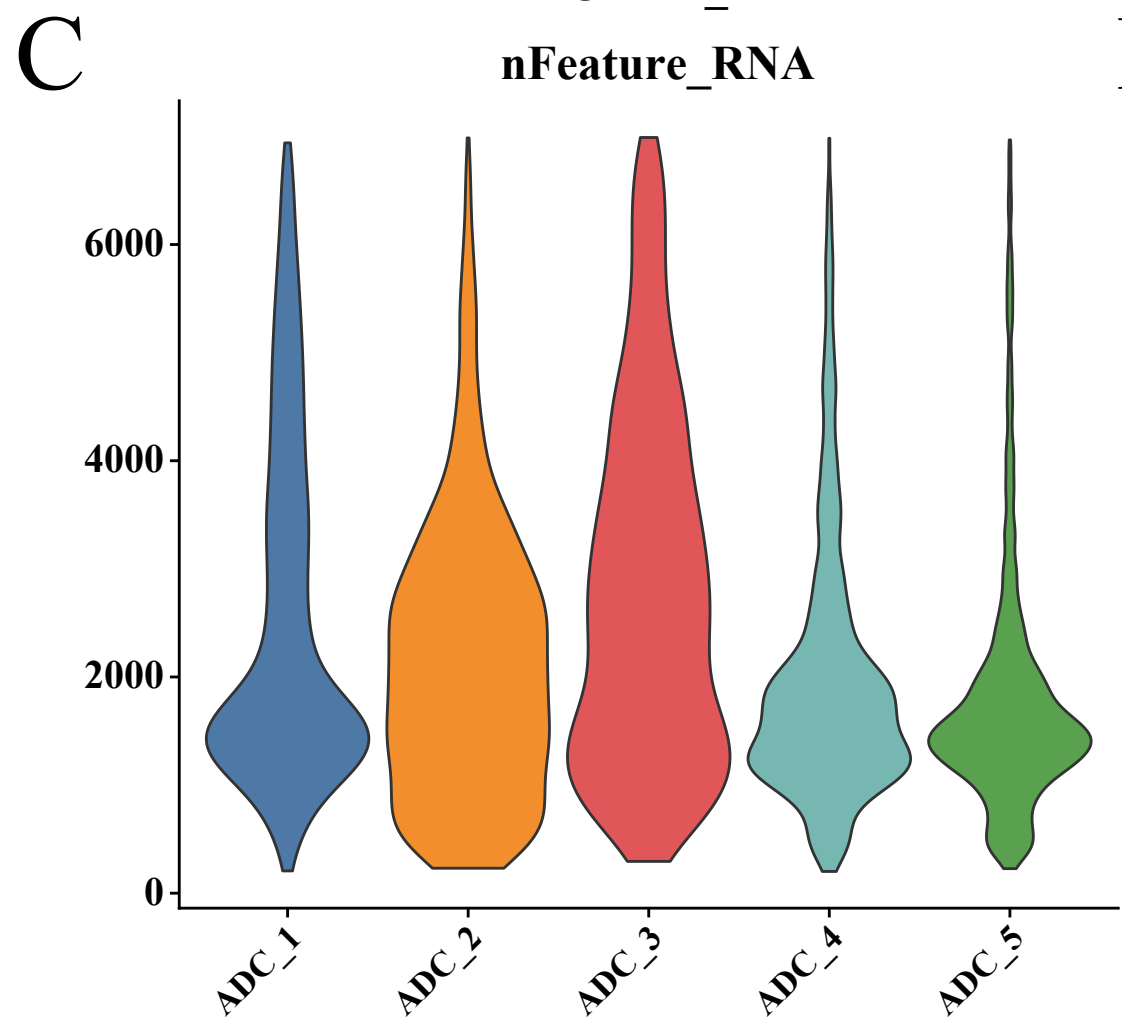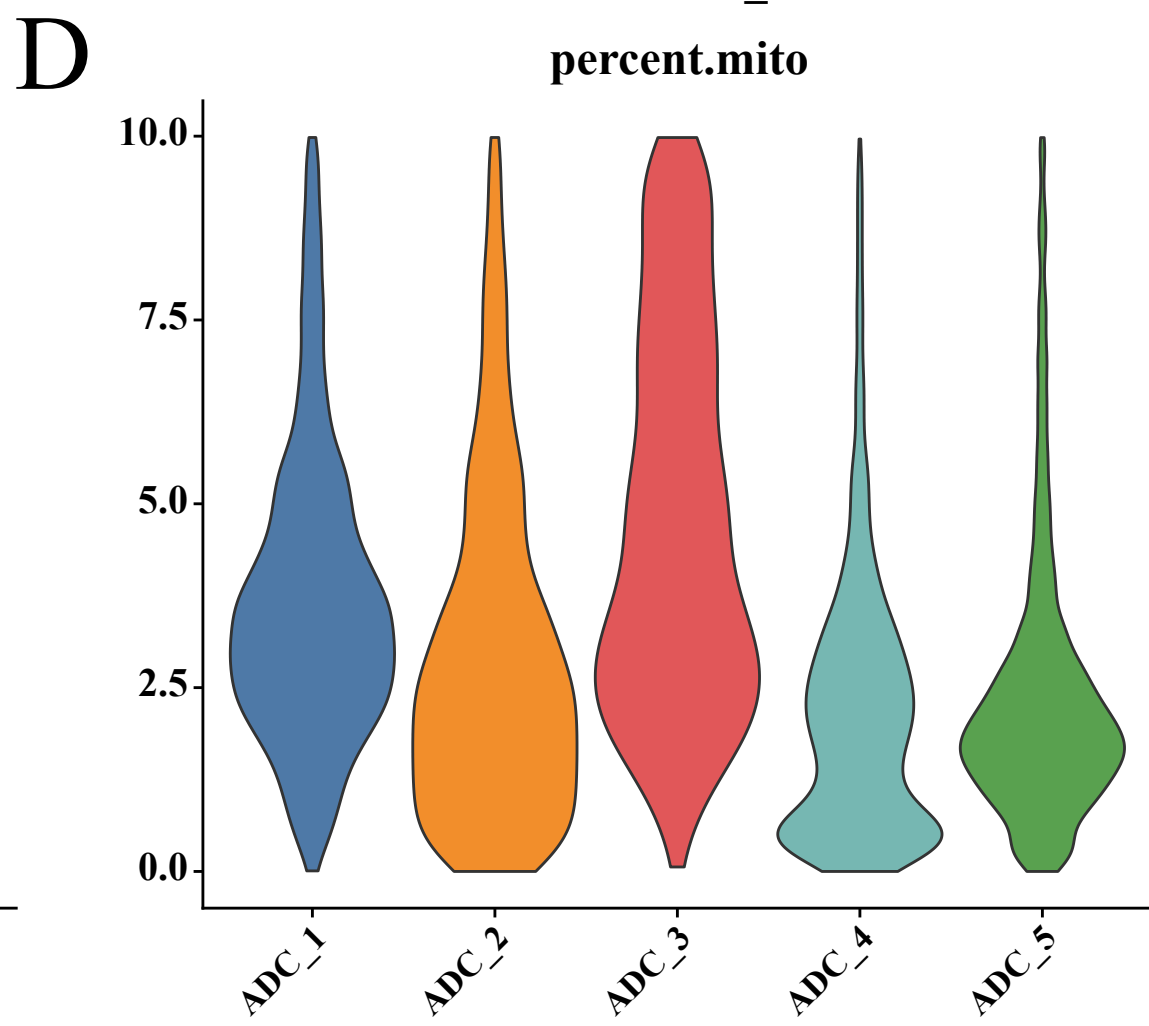

Supplement: Supplemental Information 1 — (A) The UMAP of cell cluster in different samples. (B) The UMAP of cell clusters in HPV (+) and HPV (-) groups. (C) The gene number of the screened cells. (D) The mitochondrial ratio of screened cells. [file peerj-12-18158-s001.pdf]
